# Normalization of short-chain fatty acid concentration by bacterial count of stool samples improves discrimination between eubiotic and dysbiotic gut microbiota caused by *Clostridioides difficile* infection-

**DOI:** 10.1080/19490976.2024.2415488

**Published:** 2024-10-12

**Authors:** Anna Sayol-Altarriba, Andrea Aira, Anna Villasante, Rosa Albarracín, Joana Faneca, Gregori Casals, José Luis Villanueva-Cañas, Climent Casals-Pascual

**Affiliations:** a Faculty of Medicine and Health Sciences, University of Barcelona (UB), Barcelona, Spain; b ISGlobal, Barcelona, Spain; c Department of Clinical Microbiology, Centre for Biomedical Diagnosis, Hospital Clínic de Barcelona, Barcelona, Spain; d Centro de Investigación Biomédica en Red (CIBERINFEC), Barcelona, Spain; e Department of Biochemistry and Molecular Genetics, Centre for Biomedical Diagnosis, Hospital Clínic de Barcelona, IDIBAPS, Barcelona, Spain; f Molecular Biology CORE, Centre for Biomedical Diagnosis, Hospital Clínic de Barcelona, Barcelona, Spain

**Keywords:** Short-chain fatty acids (SCFAs), *Clostridioides difficile*, gut, intestinal microbiota, dysbiosis, fecal microbiota transplantation (FMT)

## Abstract

Short-chain fatty acids (SCFAs) represent a cornerstone of gut health, serving as critical mediators of immune modulation and overall host homeostasis. Patients with dysbiosis caused by *Clostridioides difficile* infection (CDI) typically exhibit lower SCFAs levels compared to healthy stool donors and, thus, the concentration of SCFAs has been proposed as a proxy marker of a healthy microbiota. However, there is no consistency in the methods used to quantify SCFAs in stool samples and usually, the results are normalized by the weight of the stool samples, which does not address differences in water and fiber content and ignores bacterial counts in the sample (the main component of stool that contributes to the composition of these metabolites in the sample). Here, we show that normalized SCFAs concentrations by the bacterial count improve discrimination between healthy and dysbiotic samples (patients with CDI), particularly when using acetate and propionate levels. After normalization, butyrate is the metabolite that best discriminates eubiotic and dysbiotic samples according to the area under the receiver operating characteristic (ROC) curve (AUC-ROC = 0.860, [95% CI: 0.786–0.934], *p* < .0001).

## Introduction

Short-chain fatty acids (SCFAs) represent key metabolic by-products synthetized by gut bacteria through anaerobic fermentation of dietary fiber and resistant starches that have not been assimilated by the host,^1^ with proteins and peptides contributing to a lesser extent. The main SCFAs are acetic, propionic, and butyric acids, which are found in their anionic form in the human colon (acetate, propionate, and butyrate).^2^ While acetate is produced by most of the gut bacterial population, the synthesis of propionate and butyrate is limited to certain groups of colonic bacteria through multiple pathways and cross-feeding mechanisms, where intermediate and final metabolites are exchanged between different species,^3–6^ Specifically, most butyrate-producing species belong to *Ruminococcaceae* and *Lachnospiraceae*, the two predominant families of Firmicutes members found in the human colon;^6,7^ while propionate-producing species belong mainly to Bacteroidetes, the Negativicutes class of Firmicutes, and the *Lachnospiraceae* family.^6,8^

These metabolites play diverse roles crucial for both gut and systemic health. Among several functions, they contribute to maintaining gut homeostasis and integrity,^9^ influence whole-body metabolism,^5^ act as an energy source of colonocytes (particularly butyrate),^4^ promote mucus production in a dose-dependent manner,^10,11^ exhibit antimicrobial, anti-inflammatory, and antitumorigenic activities,^12,13^ and modulate the immune system.^13^ Considering their beneficial roles and implications in diseases characterized by alterations in the microbiome, SCFAs could serve as promising biomarkers. Such diseases range from intestinal pathologies such as inflammatory bowel diseases (IBDs)^14,15^ or patients with *Clostridioides difficile* infections (CDI), ^16–18^ to metabolic diseases like obesity,^19–21^ cardiovascular diseases,^22,23^ and colorectal cancer.^24,25^ In addition, the implementation of fecal microbiota transplantation (FMT) for other diseases beyond CDI depends on the proper characterization of the ideal gut microbiota for donor selection, which is still unknown.^26^ The increase in SCFAs production has been proposed to be crucial in the prevention and treatment of many diseases, although long-term interventional studies in humans are still lacking.^27,28^

The main challenge in ascertaining the specific role of SCFAs in these conditions is the standardization and robustness of quantification methods, which would enable direct comparisons across studies. The predominant instrumental methods include gas chromatography coupled with mass spectrometry (GC/MS), high-performance liquid chromatography coupled to tandem mass spectrometry (HPLC-MS-MS), nuclear magnetic resonance (NMR), and capillary electrophoresis (CE).^29–31^ Although the quantification of SCFAs by GC/MS is the most common method, different studies incorporate slight variations. For instance, pre-treatment processing of the samples differs among studies (centrifugation, filtration, acidification, derivatization),^32,33^ making the comparison across studies impossible.

Despite the evident benefits of using an internal standard (IS) to overcome the possible loss of the sample during the analysis, its implementation is not common. When used, there is a lack of consensus in the kind of IS to choose, which can range from deuterated (H^2^ labeled) SCFAs,^34–36 13^C labeled SCFAs,^29,37^ or other non-majority SCFAs found in very low quantities in stool samples.^22^ A good internal standard must provide a similar signal and behavior during the quantification and be absent in the samples to analyze. This can be equally achieved with the use of deuterated and^13^C-labeled SCFAs, as they all use isotope-labeled forms of the analytes present in the sample.

Another challenge in quantifying SCFAs lies in the units used to express the results. Most studies quantify SCFAs directly (µM or µg/mL),^35,36,38^ per gram of fresh weight (µM/g)^33,39^ or dry weight (mg/g).^22^ Feces constitute a complex mixture of undigested dietary components such as fiber and other non-absorbed solids, water, and bacteria, exhibiting variability in the quantity of these constituents.^40^ The fiber and water content are interdependent, with higher fiber content leading to increased water retention, a phenomenon known as water holding capacity. Moreover, undigested carbohydrates increase the total stool weight.^41^ Although when considering the dry weight differences due to the water content of the samples are eliminated, differences in the fiber content are still not addressed. Furthermore, the main component of feces and responsible for SCFA production are bacteria (up to 55% of dry mass),^42,43^ which are not considered in these studies.

Altogether, this suggests that to properly differentiate between healthy and dysbiotic patients through SCFA concentrations, normalization by bacterial count is a necessity. In this study, we propose a novel normalization methodology and evaluate it by comparing SCFA levels between healthy donors from the Stool Bank of Hospital Clinic of Barcelona and dysbiotic patients with CDI. Optimization of the methods for SCFAs quantification through GC/MS and bacterial count normalization have also been proposed. Finally, we assess the bacterial and metabolomic stability over time of stool samples from patients with CDI as there were differences in the collection process between samples from healthy donors and dysbiotic patients.

## Materials and methods

### Sample selection and conservation

Stool samples from healthy donors (*n* = 115) were collected by the Stool Bank of Hospital Clinic of Barcelona (2021–2022) on the day of the donation and conserved at −80°C until its processing. Donors were selected following a principle of exclusion after an interview and an extensive screening which consisted of several laboratory analyses of blood and stool.^26^ Samples from patients with dysbiosis caused by CDI (*n* = 40) were collected from stool leftover (surplus) samples at the Department of Clinical Microbiology of Hospital Clínic of Barcelona. These were samples with a positive PCR test for one or both *C. difficile* toxins (Tox B/binary), prioritizing recent samples (25 out of the 40 samples with CDI used were processed within <96 h after collection) and with a Bristol Stool Form Scale above 4.^44^ Stools collected from patients were stored at 4°C until their first processing. All samples used for the study were anonymized using an alphanumeric code. The study was approved by the Ethics Committee of Research with medicines (CEIm) of Hospital Clínic of Barcelona (Ref. HCB/2024/0239). All healthy donors provided appropriate informed consent. Requirement consent from CDI patients was waived by the ethics committee as the samples used came from surplus of the hospital’s Clinical Microbiology Department, without having access to any clinical or personal data from these patients.

### Bacterial count of stool samples by flow cytometry

#### Optimization of the bacterial count methodology

To optimize the bacterial count procedure, we performed a serial dilution of six stool samples from healthy donors. First, a 1:10 (w/v) dilution from the direct stool sample was prepared. From this, several serial dilutions were made (1:100; 1:1,000; 1:5,000, and 1:10,000 v/v). Sterile water for injectable preparations (B. Braun, Melsungen, Germany) was used as a diluent, with a final volume of 5 mL in all of them. Each dilution was homogenized with the vortex, and if there were large particles in the sample, these were avoided by taking the total volumes for the dilutions in small amounts.

The bacterial count (bacteria/µL) of the three last dilutions (1:1,000; 1:5,000 and 1:10,000) was measured by flow cytometry using the body fluid mode (UF-400, Sysmex Co., Kobe, Japan) and with 5 technical replicates per sample (*n* = 6).

#### Bacterial count

After the optimization of the procedure, the bacterial count (bacteria/µL) of stool samples was quantified using the 1:5,000 (v/v) dilution for samples from healthy donors, and the 1:1,000 (v/v) dilution for samples from dysbiotic patients with CDI. After the recount, the bacterial concentration of the original sample was calculated for the two groups.

### SCFAs quantification by GC/MS and normalisation by bacterial count and fresh weight

SCFAs were analyzed by GC/MS as previously described by Zhang et al. 2019, with minor modifications:

#### Acidification of stool samples

~100 mg of stool was suspended in sterile acidified water with hydrochloric acid (HCl) (acidified water with HCl 12.06 N; until pH = 2.5) at a final concentration of 100 mg of stool/mL. The suspension was vigorously vortexed until the sample was completely homogenized. Next, the tubes were centrifuged at 13,000 rpm for 3 min. Five hundred microliters of the supernatant was transferred to a fresh tube and stored at −20°C until the day of the GC/MS analysis (~2 months).

#### SCFAs extraction

Tubes containing the acidified supernatant were placed at room temperature until completely defrosted. Then, they were centrifuged at 14,000 rpm for 10 min, and 100 µL of the sample supernatants was placed into a fresh 0.6 mL tube. In the same tube 70 µL of ultrapure H_2_O, 30 µL of internal standard (IS) (propionic acid-d_3_, 0.5 mg/mL) (Toronto Research Chemicals, North York, Canada), 200 µL of HCl 5 M, and 200 µL of anhydrous diethyl ether (1:1, v/v) were added.

Tubes were vigorously vortexed for 1–2 min and centrifuged at 14,000 rpm for 15 min for the SCFAs extraction. After the centrifugation, 50 µL of the upper phase of the solution (diethyl ether layer containing SCFAs) was transferred into a fresh glass vial of injection. Samples were then re-extracted with 150 µL of diethyl ether, and 100 µL of the upper phase of the solution were transferred into the vial of injection (containing the 50 µL of the first extraction). Fifty microliters of diethyl ether and 20 µL of BSTFA and trimethylchlorosilane (TMCS) (99:1) (Merck, Darmstadt, Germany) were added. Vials of injection were incubated for 2 h at 40°C for derivatization.

For the quantification, standard curves were prepared with increasing concentrations of standards of acetate, propionate, and butyrate (Merck, Darmstadt, Germany) as calibrators. The blank samples and the calibrators were processed following the same procedure as the fecal samples (extraction with anhydrous diethyl ether and HCl, centrifugation, and derivatization).

#### GC/MS analysis

Samples were placed at the GC/MS instrument (GCMS-QP210 Ultra, Shimadzu Co., Kyoto, Japan) with a Sapiens 5-MS capillary column (30 m × 0.25 mm × 0.25 µm) (Teknokroma, St. Cugat del Vallès, Spain). The injector, ion source, and GC/MS interface temperatures were 280°C, 250°C, and 280°C, respectively. For the analysis, the flow rate of the helium carrier gas was kept at 1 mL/min, and 1 µL of the derivatized sample was injected with a 3-min solvent delay time and split ratio of 10:1. The initial column temperature was 40°C and held 2 min, increased until 150°C at the rate of 15ºC/min and held 1 min, and then increased to 300°C at the rate of 80ºC/min and kept at this temperature for 3 min. The ionization was carried out in the electron impact (EI) mode at 70 eV.

The identification of compounds was corroborated by the injection reference standards of acetic (purity ≥99.8%), propionic (purity ≥99.5%), and butyric acid (purity ≥99%) (Sigma-Aldrich, St. Louis, MO, USA) in scan mode, and the comparison of the retention time and corresponding MS spectra with data from the samples. Compounds were quantified in the selected ion monitoring (SIM) mode using the target ion. The target ion (*m/z*) of acetic, propionic, propionic-d_3,_ and butyric acids were 117, 131, 134, and 145, respectively (Supplementary Table S1, Supplementary Figure S1).

Calibration curves with reference standards were linear (R^2^ ≥0.99) up to 10,000 µM. No carry-over was observed in blank samples that were injected after these high concentration standards. The inter-assay coefficient of variations of a pool of samples was <8%, and the recoveries of spiked samples were 88–114%. Interday accuracy and precision values of the calibration curve standards can be found in the supplementary materials (Suplementary Table S2).

#### Normalization of SCFAs concentrations

For the normalization of SCFAs levels by bacterial count, the bacterial concentration results of the original stool sample (bacteria/µL) were ranked according to the maximum value for each population of samples separately (each bacterial count result was divided by the maximum). Then, the not-normalized SCFA concentration of each sample was multiplied by the corresponding ranked value of the bacterial count. With this, the results of the SCFAs concentration were adjusted by the bacterial count of the stool sample. For the normalization by fresh weight of the sample, not-normalized SCFA concentrations were divided by the fresh weight of each sample (the exact weight of the stool sample taken for the acidification, around 100 mg).

### Bacterial and metabolomic stability of stool samples from patients with CDI

To discard possible changes in the bacterial count and the metabolomic analysis of samples kept at 4°C, random stool samples from patients with CDI were assessed during four consecutive weeks (1 month) (*n* = 3). At each time point, both the bacterial count and the acidification of the stool sample were assessed, as outlined in the preceding sections. Between the different assessments, samples were kept at 4°C in their original container. The supernatant of the stool acidification of each timepoint was kept at −20°C and processed on the day of the GC/MS analysis with the rest of the samples.

### Statistical analysis

Statistical analysis of the bacterial count and SCFA concentrations was done using R through RStudio Software (RStudio: Integrated Development Environment for R, Boston, MA, USA) (version 4.3.1). To determine if there were significant differences in the bacterial count and the SCFA levels between the two groups (healthy donors and dysbiotic patients with CDI), we applied the U of Mann–Whitney test. For the assessment of the bacterial and metabolomic stability of stool samples over time, we applied the Friedman test. Receiver operating characteristic (ROC) and precision-recall (PR) curves were used to confirm the predictive capacities of SCFAs (acetate, propionate, and butyrate) in detecting healthy donors. The ROC curve parameters (AUC-ROC, 95% CI, optimum cutoff, sensitivity or True Positive Rate, specificity or True Negative Rate, and *p*-value) and pairwise comparisons for two correlated ROC curves were calculated with the pROC R package using the DeLong method.^45^ PR curves were generated using the PRROC R package.^46^

## Results

### Bacterial count of stool samples by flow cytometry

#### Optimization of the bacterial count methodology

**Figure 1.**
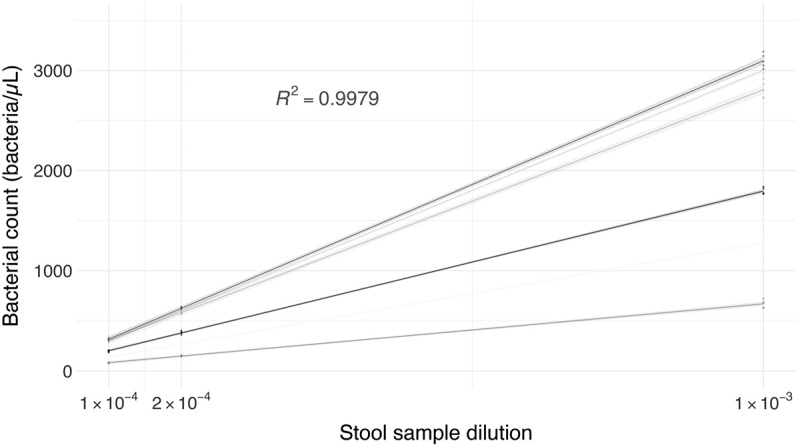
Bacterial count (bacteria/µl) of stool samples. Dilutions 1:1,000; 1:5,000, and 1:10,000 were assessed (five technical replicates per dilution). R^2^ average of 0.9979 (*n* = 6).

Curves of the optimization of the bacterial count method showed a linearity above 0.99 between serial dilutions of stool samples and the bacterial count with an average of 0.9978 (Figure 1). The average standard deviation was 50.8, 10.9, and 10.2 for the 1:1,000; 1:5,000, and 1:10,000 dilutions, respectively. Differences between the bacterial count of different donors could be observed.

#### Bacterial count

**Figure 2.**
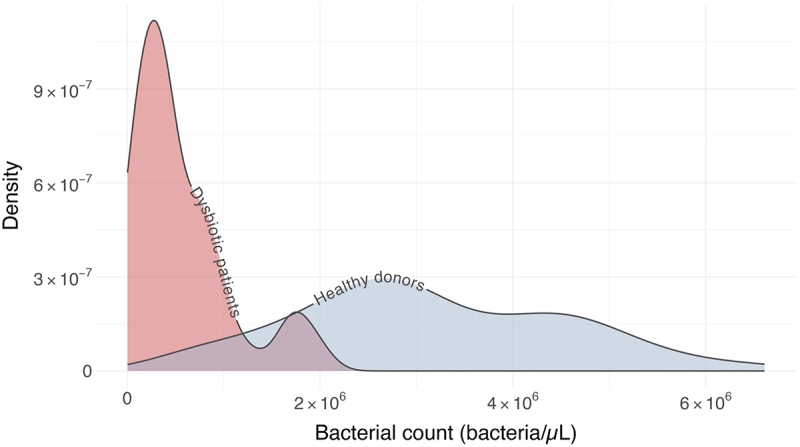
Density plot of the bacterial counts (bacteria/µl) of the original sample. Data from healthy donors (*n* = 115) (blue) and dysbiotic patients with CDI (*n* = 40) (red).

Bacterial counts of stool samples from healthy donors followed a normal distribution (Shapiro–Wilk test for normality, *p*-value = 0.1381). In contrast, data from dysbiotic patients with CDI did not follow a normal distribution (*p*-value <0.01) (Figure 2). The bacterial count showed differences between the two groups according to the U of Mann–Whitney test (*p*-value <0.01). One sample from the CDI group was considered an outlier regarding the bacterial count and was not considered in subsequent analysis nor for the normalization of SCFAs concentrations.

### SCFAs quantification by GC/MS and normalization by bacterial count and fresh weight

**Figure 3.**
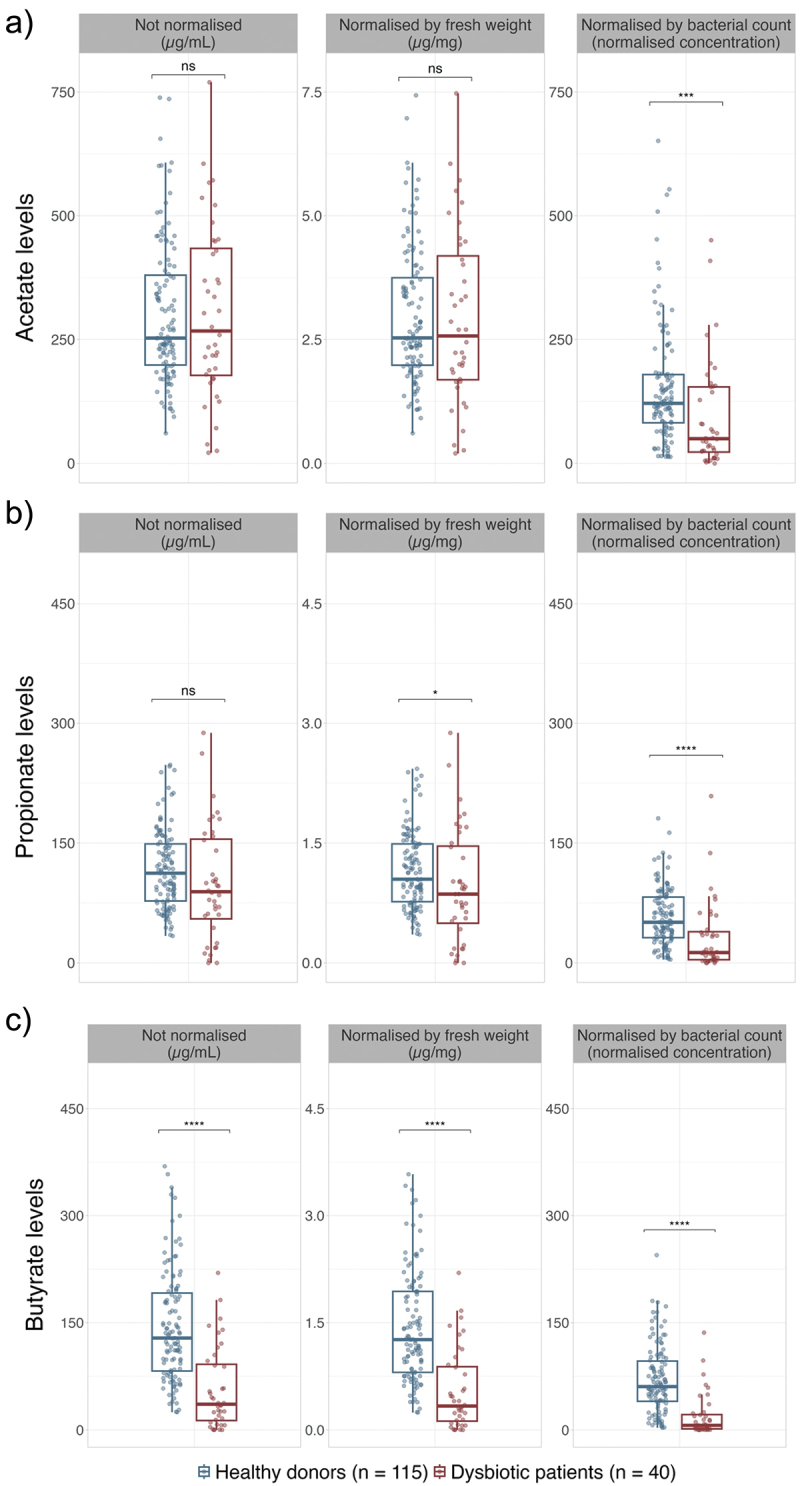
Quantification of the SCFA levels without normalization (µg/mL), normalized by fresh weight (µg/mg), and normalized by bacterial count of healthy donors (*n* = 115) (blue) and dysbiotic patients with CDI (*n* = 40) (red). a) acetate, b) propionate, and c) butyrate.

The results of the quantification of SCFA levels in stool by GC/MS measurements showed that significant differences in butyrate levels can be detected between healthy donors and dysbiotic patients with CDI regardless of the normalization of the concentrations (*p*-values <0.0001) (Figure 3c). In contrast, differences between the two populations cannot be detected if acetate and propionate levels are not normalized. Despite the normalization by fresh weight (µg/mg) showing significant differences in the case of propionate levels (*p*-value = 0.0333) (Figure 3b), differences in acetate levels are not detected (Figure 3a). Moreover, when normalizing acetate and propionate concentrations by bacterial count (µg/mg), greater differences in these SCFAs concentrations between healthy donors and dysbiotic patients with CDI are detected (Table 1, Figure 3).

**Table 1.** Quantification of SCFAs in human feces.

	Healthy donors (n = 115)			Dysbiotic patients (n = 40)			
Analyte	Median ± IQR	Q1	Q3	Median + IQR	Q1	Q3	*p-*value^a^
SCFA concentration without normalization (µg/mL)							
Acetate	252.98 ± 181.30	198.66	379.95	267.28 ± 256.29	177.71	434.00	0.8980
Propionate	112.34 ± 71.41	77.51	148.91	89.15 ± 99.73	55.18	154.92	0.0614
Butyrate	128.59 ± 109.11	82.46	191.57	36.00 ± 78.64	13.22	91.86	<0.0001
SCFA concentration normalized by fresh weight (µg/mg)							
Acetate	2.53 ± 1.76	1.98	3.75	2.57 ± 2.50	1.69	4.19	0.6540
Propionate	1.05 ± 0.72	0.77	1.49	0.86 ± 0.97	0.50	1.46	0.0333
Butyrate	1.26 ± 1.13	0.81	1.94	0.33 ± 0.76	0.12	0.89	<0.0001
SCFA concentration normalized by bacterial count							
Acetate	121.32 ± 97.32	82.08	179.40	49.93 ± 131.56	22.93	154.49	0.0002
Propionate	50.90 ± 50.86	31.56	82.42	12.96 ± 35.04	3.99	39.02	<0.0001
Butyrate	60.69 ± 56.30	40.04	96.34	6.41 ± 19.98	1.54	21.52	<0.0001

^a^*p-*value of the U of Mann-Whitney test

### Bacterial and metabolomic stability of stool samples from patients with CDI

The bacterial count and the metabolomic stability of stool samples were assessed with the Friedman test, which assesses whether there are any statistically significant differences between the distribution of three or more paired groups with a nonparametric distribution. Results over the four time points assessed (1 month) (*n* = 3) showed no significant differences in the bacterial count (*p*-value = 0.45), and levels of acetate, propionate, and butyrate (*p*-values >0.01 for all SCFAs).

### ROC curve analysis for detecting healthy donors and SCFAs levels distribution

**Figure 4.**
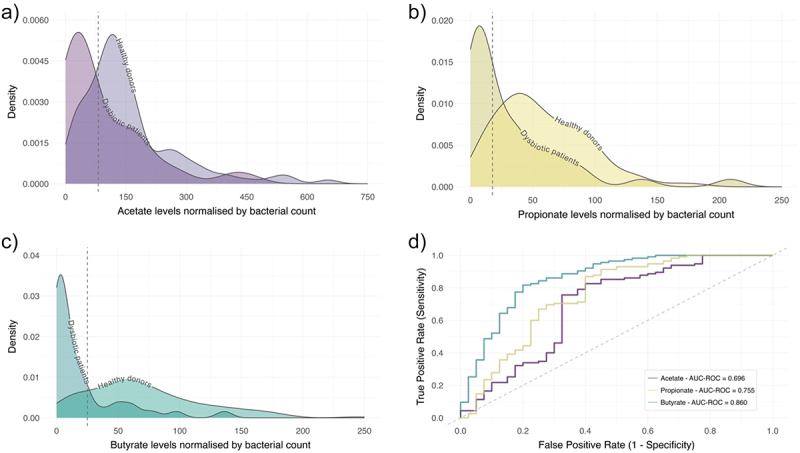
Distribution of SCFAs levels normalized by bacterial count and ROC curve analysis for comparison of SCFAs levels in detecting healthy donors. a) acetate (purple), b) propionate (yellow), and c) butyrate (green); d) ROC curve for acetate (AUC-ROC = 0.696), propionate (AUC-ROC = 0.755) and butyrate (AUC-ROC = 0.860). In the density plots the dashed lines indicate the optimal cutoffs calculated in the ROC curve, in the ROC curve the dashed line is the random classifier. Healthy donors (*n* = 115) and dysbiotic patients with CDI (*n* = 40).

The distribution of SCFAs levels normalized by bacterial count did not follow a normal distribution in any case (*p*-value <.01) (Figure 4a–c). ROC curves showed that butyrate levels with the normalization by bacterial count might be better for identifying healthy donors than the other SCFAs. The areas under the ROC curves for the different SCFAs concentration normalized by bacterial count were the following: acetate (AUC-ROC = 0.696, 95% CI 0.588–0.804), propionate (AUC-ROC = 0.755, 95% CI 0.656–0.855), and butyrate (AUC-ROC = 0.860, 95% CI 0.786–0.934), being the *p*-value <0.001 in all the cases. The optimum cutoff values for the normalized levels were determined by the ROC curves to identify healthy donors and were >81.077 for acetate (sensitivity = 75.7%, specificity = 67.5%), >17.644 for propionate (sensitivity = 87.0%, specificity = 60.0%), and >25.019 for butyrate (sensitivity = 81.7%, specificity = 80.0%) (Table 2, Figure 4d).

**Table 2.** ROC curve parameters and optimum cutoff values of SCFAs concentrations normalized by bacterial count.

Parameter	AUC-ROC	95% CI		*p-*value	Optimum cutoff	Sensitivity	Specificity	Precision
		Lower limit	Upper limit					
Acetate	0.696	0.588	0.804	<0.001	>81.077	0.757	0.675	0.870
Propionate	0.755	0.656	0.855	<0.0001	>17.644	0.870	0.600	0.862
Butyrate	0.860	0.786	0.934	<0.0001	>25.019	0.817	0.800	0.922

The comparison between correlated ROC curves showed significant differences in all cases. Being differences between acetate and butyrate ROC curves the most significant ones (*p*-value <.0001), followed by propionate and butyrate (*p*-value <.0001), and acetate and propionate (*p*-value <0.05).

Additionally, a ROC curve combining the three SCFAs according to the molar ratio of acetate:propionate:butyrate in the human colon (60:20:20 respectively)^2^ was tested. This curve showed poorer results than the propionate and butyrate ones (AUC-ROC = 0.720, 95% CI 0.617–0.824) (Supplementary Figure S2). PR-curves corroborated that butyrate presents a higher classification performance than acetate and propionate (Suplementary Figure S3).

## Discussion

SCFA is likely to be among the most predictive quality markers in human microbiota samples. Therefore, establishing robust quantification protocols is essential for progressing not only fecal microbiota transplantation (FMT) procedures but also for the characterization of the gut microbiome in both health and disease. Current challenges in this area include variability in GC/MS pre-treatment protocols and the need to normalize SCFA levels. In this study, we demonstrate that the normalization of SCFA concentrations by bacterial counts in stool samples allows to distinguish healthy donors from dysbiotic patients with CDI.

For SCFAs quantification, we used GC/MS as a reference analytical method, with a pre-treatment based on acidification followed by an organic solvent extraction, centrifugation, and derivatization. With this pre-treatment, a higher thermal stability, volatility, and sensitivity, and a greater purification of the sample and moisture removal are achieved.^32,33^ Despite these evident advantages, the process is more time-consuming (has additional steps), expensive (requires more reagents), and may lead to analyte loss during the protocol.^32^ To overcome possible SCFA loss during the extraction and the measurement by GC/MS, we additionally used a deuterated IS, which was not included in the well-validated original method by Zhang et al.^33^ However, as the IS was not added until extraction with diethyl ether, potential losses of the sample during the acidification, freezing, and thawing steps are still possible.

To prevent the interference of differences in water and fiber content of stool samples in SCFAs quantification, we developed a normalization by bacterial count. To achieve this, an optimal method for bacterial quantification is crucial. Our method of quantification by flow cytometry shows excellent linearity in all the samples (>0.99), with an average correlation coefficient of 0.9978 (Figure 1). Thus, any of the dilutions of stool samples assessed could be used for the bacterial quantification. Considering the significant differences between the bacterial content of stool samples between healthy donors and dysbiotic patients with CDI (Figure 2), we decided to use the 1:5,000 and 1:1,000 dilutions, respectively. With this, the presence of particles that could obstruct the flow cytometry was avoided, and the bacterial quantification was not compromised. Possible adherence of bacteria to particles present in the stool sample was not assessed. To our understanding, this is an uncontrolled variable intrinsic to studies that involve bacteria in stool samples, and which is in turn correlated with other variables such as diet or the digestion process.

Only when the results are normalized by bacterial count significant differences in SCFA concentrations be found in stool between healthy donors and dysbiotic patients with CDI. Although the normalization by fresh weight of the stool sample allows discriminating between the two groups in the case of propionate and butyrate levels, differences are lower than when normalizing by bacterial count (Table 1, Figure 3b,c).

Even though the use of bacterial counts for normalization in stool samples enhances the ability to distinguish between the two groups, butyrate levels consistently differentiate healthy donors from dysbiotic patients with CDI, irrespective of whether results are normalized (Figure 3c). These findings are consistent with the fact that the bacterial genera *Faecalibacterium, Roseburia, Anaerostipes*, and *Coprococcus* contain the main butyrate-producing species and show decreased abundances in stool samples of patients with CDI.^16,17^ In addition, *in vitro* studies have shown that butyrate can inhibit the growth of multiple *C. difficile* strains^47–49^ probably due to its role in enhancing the barrier function in the gut through anti-inflammatory and antimicrobial activities and the promotion of mucin production.^27,50,51^ In murine models, it has also been observed that diets that enhance butyrate production reduce the burdens caused by CDI.^48^ Altogether, these results suggest that to properly proliferate and colonize the gut, *C. difficile* might require a previous depletion of butyrate-producing species, which may explain why significant differences in butyrate levels are observed whether results are normalized or not.

Unlike propionate and butyrate production, which is limited to certain groups of bacteria, acetate can be synthetized by most colonic bacteria.^4–6^ This may explain why acetate levels have a limited discriminatory capacity to differentiate between healthy donors and dysbiotic patients with CDI.

Our findings reveal the importance of normalizing SCFA concentrations by the number of bacteria, the main component of stool samples, and being in charge of SCFA production.^2,42,43^ Although normalization by dry weight^22^ or fresh weight^33,39^ has been previously applied in other studies, our findings show that the normalization of SCFAs concentrations by fresh weight of the sample has a limited capacity for differentiating among healthy donors and dysbiotic patients with CDI, especially in the case of acetate and propionate (Figure 3a,b). Thus, to properly distinguish between healthy and dysbiotic patients with CDI using acetate, propionate, and butyrate concentrations, normalization by bacterial count should be applied. These findings are also consistent with the fact that dysbiosis, although controversial, exists, and SCFA may be a good proxy to detect so.

Likewise, ROC curves of the SCFAs levels normalized by bacterial count confirm that butyrate levels have an excellent capacity to identify healthy donors (AUC-ROC = 0.860, *p*-value <0.0001), compared to propionate (AUC-ROC = 0.755, *p-*value <0.0001) or acetate (AUC-ROC = 0.696, *p-*value <0.001). According to the DeLong method, the suggested cutoff for butyrate bacterial normalized levels for discriminating between healthy donors and dysbiotic patients is >25.019, with a precision of 92.2%, and a sensitivity and specificity of 81.7% and 80.0%, respectively (Table 2, Figure 4c).

This study presents several limitations. Firstly, normalization by the dry weight of stool samples was not performed and could not be compared to the other normalizations methods of SCFA concentrations. Secondly, unlike samples from healthy donors, samples from patients with CDI could not be immediately frozen after arriving at the laboratory and were kept at 4°C until the acidification. This was due to the provenance of the samples, as they came from care surpluses. Although we cannot completely exclude the temperature-dependent effect on SCFA quantification, the analysis of the bacterial count and SCFA levels of three random samples over time (1 month) shows no significant differences between time points according to the Friedman test. These results suggest that although stool samples from patients with CDI could not be immediately frozen, the bacterial and metabolomic stability is maintained during the time of preservation. This assessment was not performed with healthy donor samples as the immediate freezing of the samples at −80°C is considered the reference method.^30^

In addition, as CDI represents an acute clinical picture, the dysbiosis might be more severe than in other gastrointestinal diseases with a chronic course, such as IBDs. Thus, future studies should focus on the assessment of samples with intermediate forms of dysbiosis, to confirm if SCFA levels can be used to differentiate between these patients and the healthy population.

## Conclusions

The normalization of SCFAs concentrations by bacterial count allows a proper differentiation between healthy donors and patients with dysbiosis caused by CDI using acetate, propionate, or butyrate levels. Without the normalization or normalizing by fresh weight of the stool sample, differences are not observed or to a lower degree. Butyrate levels normalized by bacterial count have the highest predictive capacity to distinguish among healthy donors and dysbiotic patients with CDI, which was confirmed by ROC curves (AUC-ROC = 0.860).

## Supplementary Material

Supplemental Material

## Data Availability

The data and code required to reproduce the analysis presented in this paper are available in the following GitHub repository: https://github.com/CDB-coreBM/Normalisation_SCFAs.
