# Supplementary material for: Normalization of short-chain fatty acid concentration by bacterial count of stool samples improves discrimination between eubiotic and dysbiotic gut microbiota caused by Clostridioides difficile infection-
Source: Gut Microbes. 2024 Oct 12;16(1):2415488. doi: 10.1080/19490976.2024.2415488 (PMC11485779; doi:10.1080/19490976.2024.2415488)
Supplement: Supplemental Material [file KGMI_A_2415488_SM9640.zip › Revised_supplementary_material_clean.docx]

## Supplementary materials for

## **“Normalisation of short-chain fatty acid concentration by bacterial count of stool samples improves discrimination between eubiotic and dysbiotic gut microbiota caused by *Clostridioides difficile* infections”**

Anna Sayol-Altarriba^a,b,c^, Andrea Aira^b,c,d^, Anna Villasante^c^, Rosa Albarracín^c^, Joana Faneca^e^, Gregori Casals^e^, José Luis Villanueva-Cañas^f,┼^ and Climent Casals-Pascual^a,b,c,* ┼^

^a^Faculty of Medicine and Health Sciences, University of Barcelona (UB), Barcelona, Spain.

^b^ISGlobal, Barcelona, Spain.

^c^Department of Clinical Microbiology, Centre for Biomedical Diagnosis, Hospital Clínic de Barcelona, Barcelona, Spain.

^d^Centro de Investigación Biomédica en Red (CIBERINFEC), Barcelona, Spain.

^e^Department of Biochemistry and Molecular Genetics, Centre for Biomedical Diagnosis, Hospital Clínic de Barcelona, IDIBAPS, Barcelona, Spain.

^f^Molecular Biology CORE, Centre for Biomedical Diagnosis, Hospital Clínic de Barcelona, Barcelona, Spain.

^┼^Contributed equally

*Corresponding author. Emaill: [ccasals@clinic.cat](mailto:ccasals@clinic.cat) (CCP).

**SUPPLEMENTARY TABLES**

| **Supplementary Table 1.** Retention time and target m/z values of the SCFAs and internal standard | | |
| --- | --- | --- |
| **Analytes** | **Retention time (min)** | **Target Ion (*m/z*)** |
| Acetate | 3.20 | 117 |
| Propionate | 4.40 | 131 |
| Proptionate-d_3_^a^ | 4.35 | 134 |
| Butyrate | 5.45 | 145 |
| ^a^Internal standard (IS) | | |

| **Supplementary Table 2.** Interday accuracy and precision values of the calibration curve standards (n = 6 independent calibration runs). A: Accuracy; RSD: Relative standard deviation. | | | | | | | | |
| --- | --- | --- | --- | --- | --- | --- | --- | --- |
| **GC/MS method** | | | | | | | | |
| **Acetate** | | | **Propionate** | | | **Butyrate** | | |
| **µg/mL** | **A (%)** | **RSD (%)** | **µg/mL** | **A (%)** | **RSD (%)** | **µg/mL** | **A (%)** | **RSD (%)** |
| 300.0 | 99.0 | 1.3 | 370.0 | 99.6 | 0.5 | 440.0 | 99.6 | 0.3 |
| 150.0 | 99.9 | 4.0 | 37.0 | 99.4 | 2.4 | 44.0 | 99.5 | 2.3 |
| 15.0 | 102.4 | 2.2 | 3.7 | 104.2 | 11.4 | 4.4 | 102.8 | 7.5 |

**SUPPLEMENTARY FIGURES**

**Supplementary Figure 1.**


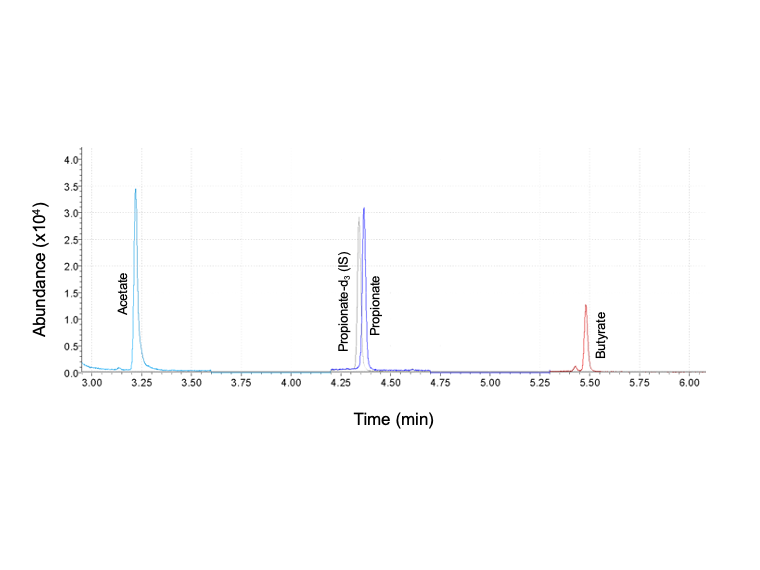


**Supplementary Figure 2.**

**
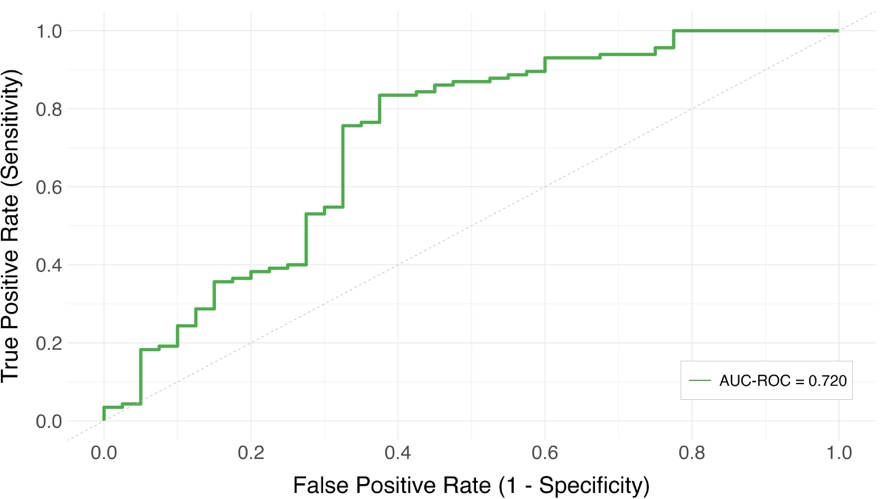
**

**Supplementary Figure 3.**

**
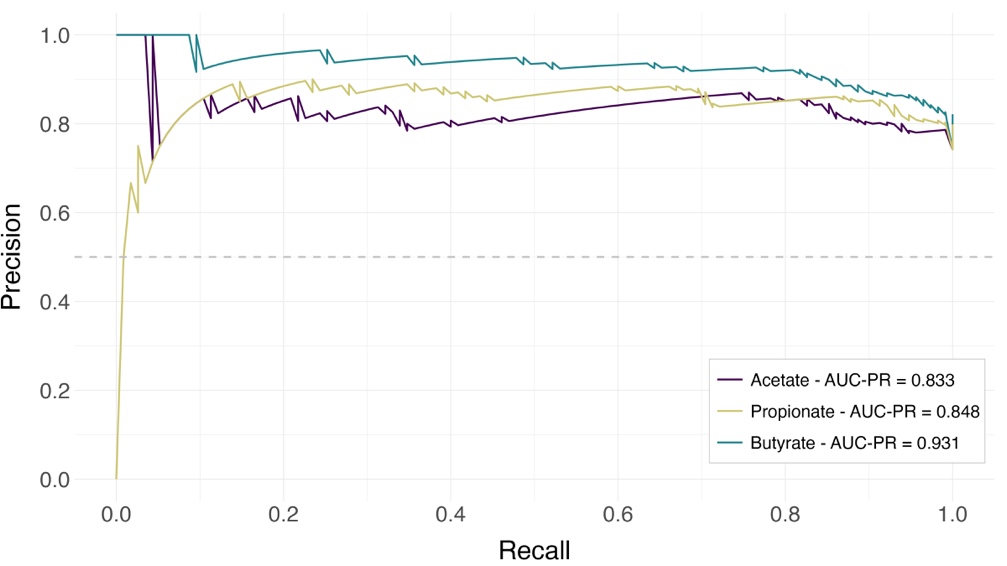
**

**SUPPLEMENTARY FIGURE CAPTIONS**

**Supplementary Figure 1.** Selected ion monitoring (SIM) chromatogram of a stool sample. Peak identification of SCFAs: acetate, propionate-d_3_ (IS), propionate, and butyrate.

**Supplementary Figure 2.** ROC curve for the combination of SCFAs according to concentration proporitons in the human gut (60% of acetate, 20% of propionate and 20 % of butyrate) (AUC-ROC = 0.720).

**Supplementary Figure 3.** PR curve for acetate (AUC-PR = 0.833), propionate (AUC-PR = 0.848) and butyrate (AUC-PR = 0.931).
